# Droplet distribution in cotton canopy using single-rotor and four-rotor unmanned aerial vehicles

**DOI:** 10.7717/peerj.13572

**Published:** 2022-06-14

**Authors:** Yanhua Meng, Yan Ma, Zhiguo Wang, Hongyan Hu

**Affiliations:** 1 School of Mechanical Engineering, Anyang Institute of Technology, Anyang, Henan Province, China; 2 Institute of Cotton Research of the Chinese Academy of Agricultural Sciences, Anyang, Henan Province, China; 3 Key Laboratory of Aviation Plant Protection, Ministry of Agriculture and Rural Affairs, Anyang, Henan Province, China

**Keywords:** Droplet distribution, UAV, Cotton canopy, Forward mode, Spraying parameters

## Abstract

Unmanned aerial vehicles (UAVs) are widely used as the sprayers for low-volume pesticide application in recent years. Droplet distribution characteristics of UAV spraying in the cotton canopy have notable effect on the biological control efficacy of the targets and the defoliation efficiency of the harvest aids. In this work, the influences on droplet distribution in the cotton canopy with respect to the flight height, forward mode, and spraying volume were evaluated by conducting the field trials during two cotton growth stages in 2020, respectively. The first field trial was performed in the cotton flowering stage and the second one was conducted in the early boll development stage. Two typical UAVs equipped with a single-rotor and four-rotor, respectively, were adopted as the spraying platforms in this work. Droplet deposition obtained by water sensitive papers (WSPs) clipped on the cotton leaves was considered as the observing metric. All cotton leaves in the canopy were divided into three groups (*i.e*., upper, middle, and bottom layers) in both trials. Furthermore, the cotton canopy was divided as eight directions to assess the droplet distribution in the canopy from different directions. The results showed that the droplet deposition varied remarkable between the treatments and in the same canopy within a treatment. The upper layer obtained higher droplet deposition than those of the middle and bottom layers and plants P4 to P8 accessed more droplets than those of the remaining sampling plants in most treatments of both trials for the two UAVs. The upper layer droplet deposition of the four-rotor UAV treatments outperformed that of the single-rotor treatments under the same operating parameters. The forward modes rarely affected the droplet distribution of the four-rotor UAV treatments but significantly influenced that of the single-rotor UAV treatments. For the single-rotor UAV spraying with “head forward”, the droplet distribution of the treatment with a flight height of 2 m was more even than that of the 1 and 3 m in the first trial. Under the same flight height, droplet deposition of the treatments with a spraying volume of 22.5 L ha^−1^ was remarkably higher than that of the 12 L ha^−1^ for both forward modes in the second trial. “Tail forward” of the single-rotor UAV treatment had better penetration at a flight height of 2 m in both trials. Therefore, for the single-rotor UAV, under a flight height of 2 m and a spraying volume of 22.5 L ha^−1^, “tail forward” was recommended for applying pesticides to control targets at the lower canopy and “head forward” was a better choice for harvest aid application. Four-rotor UAV was a suitable adoption for the harvest aid application and controlling the targets of the upper canopy. The results also indicate that the systemic pesticides are recommended for UAV spraying due to its uneven droplet distribution uniformity in the whole cotton canopy.

## Introduction

As a natural fiber, cotton is used widely in textiles globally and cotton cultivation is the main income for many farmers in the top five cotton-producing countries (Voora, Larrea & Bermudez, 2020). However, cotton plants are often attacked by various diseases and pests during the whole growth period (Xiao et al., 2019; Chi, Zhang & Dong, 2021). The damages caused by pests and diseases can result in 30–50% economic loss if no action control measure and prevention is taken (Erdogan, 2010; Xiao et al., 2019). Therefore, the spraying of pesticides is an essential practice and commonly adopted to control pests and diseases to avoid yield loss and increase agricultural productivity during the cotton cultivation season (Elhalwagy et al., 2010). Another important practice of cotton cultivation is harvest aid application, which is implemented before mechanical harvesting. The application of harvest aid aims to remove cotton leaves, inhibit regrowth, and open bolls for facilitating machine harvest all at once to avoid yield loss and assure quality (Brecke, Banks & Cothren, 2001). Harvest aids are commonly applied by foliage application using ground-based sprayers. However, ground-based sprayers are difficult to go through cotton rows due to the high-density planting pattern (Meng et al., 2019a, 2019b). High-density planting brings about serious leaf overlapping, which increases the difficulty for ground-based sprayers to pass in and out.

The application of unmanned aerial vehicles (UAVs) to spray pesticides for crop protection has been a hot issue in either industry or academia for the last decade (Lost Filho et al., 2020; Wang et al., 2020). Due to the rapid development of UAVs in crop protection, the related field application technology has been studied extensively, considering the diversity of crop canopy, pests and diseases, weather conditions, and UAV types (Kakarla, Nunes & Ampatzidis, 2019; Tsouros, Bibi & Sarigiannidis, 2019; Zhang et al., 2021). Droplet distribution in the crop canopy is a major metric to evaluate control efficacy (Qin et al., 2016; Wang et al., 2019). UAV application parameters, such as flight height, flight velocity, and spraying volume, are the key factors affecting droplet distribution and biological control efficacy on the target crops (Qin et al., 2018; Ni et al., 2021). Recently, UAVs have been developed as an essential sprayer in harvest-aids application in the cotton fields (Meng et al., 2019a, 2019b; Chen et al., 2021a) and cotton pests and diseases management (Chen et al., 2021b; Liu et al., 2021). For the purpose of defoliating cotton leaves or controlling pests and diseases using UAVs, several researchers have evaluated the impacts of spraying parameters on the droplet distribution and biological control efficacy in the cotton field. Lou et al. (2018) conducted field trials to investigate the UAV flight heights on droplet distribution and drift. The results show that the droplet uniformity, coverage, deposition, and drift of the treatment with a flight height of 2 m was higher than that of the 1.5 m. Hu et al. (2021) studied the effect of flight heights and spraying volumes on the control effect of cotton aphids and the results show that the control efficacy on the first day after treatment is highly correlated with droplet distribution. The positive correlation between control efficacy and droplet distribution was significantly affected by the flight heights and spraying volumes. Meng et al. (2019a) conducted field trials in two crop seasons to investigate the harvest aid efficiency of UAV spraying, and the results show that the optimal defoliation efficacy can be obtained under a flight velocity of 4 m s^−1^ combined with a spraying volume of 22.5 L ha^−1^.

The cotton flowering stage is a key period for controlling cotton diseases and pests. In the early stage of boll development, the vegetative growth of the cotton plants has been completed and the canopy characteristics of the plants in this stage have similarity to those periods of harvest aid application. Thus, the flowering and the boll development stages were chosen for the droplet distribution evaluation periods of the first and the second field trials, respectively. Two typical UAVs, single- and four-rotor, were employed to evaluate the effect of flight heights, forward modes, and spraying volumes on droplet distribution in the cotton canopy. The flight heights (1, 2, and 3 m), forward modes (“head forward” and “tail forward”), and spraying volumes (12.0 and 22.5 L ha^−1^) were taken into consideration as the factors affecting droplet distribution. Droplet depositions on each leaf in the first trial and in the eight directions of the canopy in the second trial were collected and assessed, respectively. The objective of this study was to evaluate droplet distribution of the UAV spraying in the cotton field aiming at improving the control efficacy and defoliation efficiency of designated stages in future practice by providing droplet distribution data under different factors mentioned above.

## Materials and Methods

### Field trial site and cotton plant

**Figure 1:**
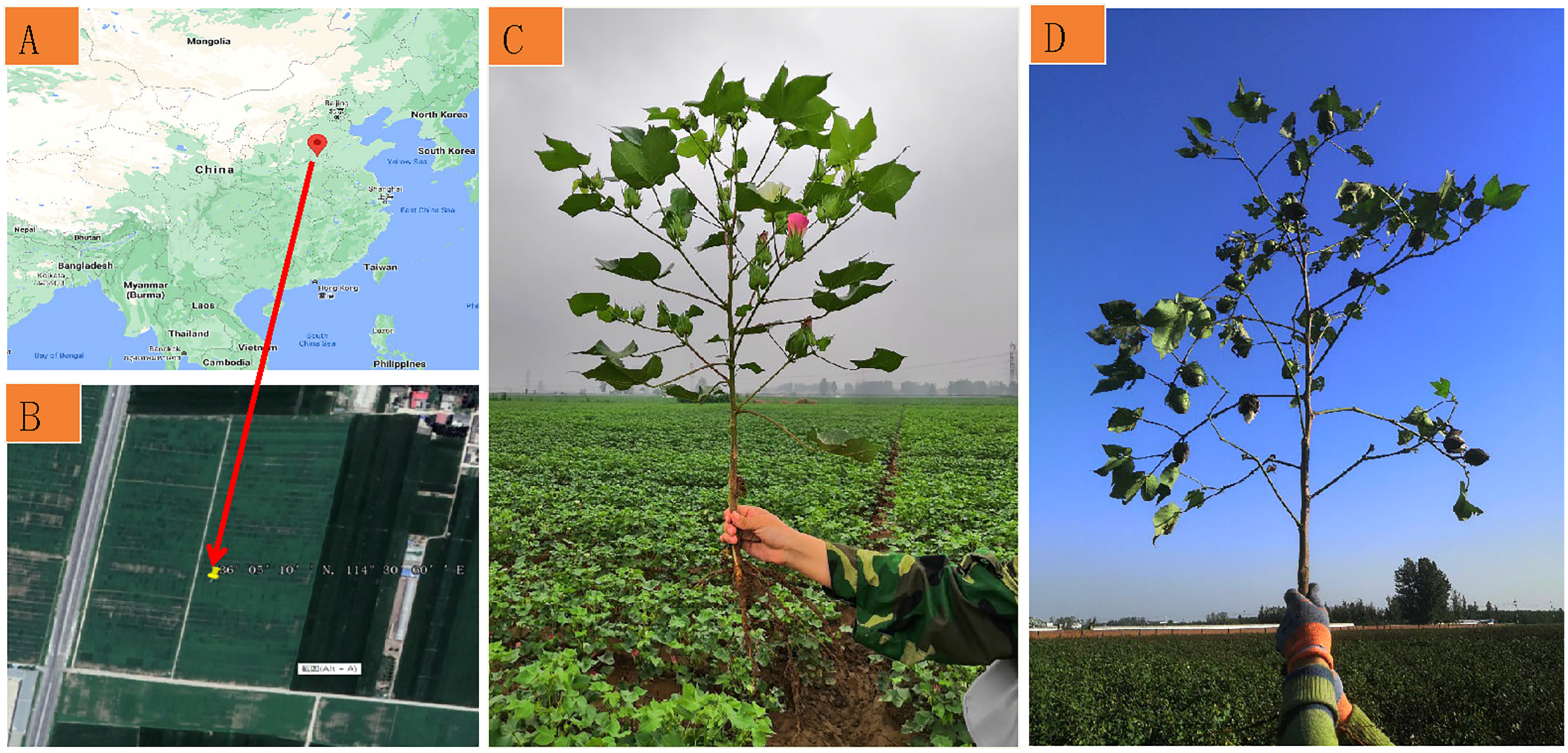
Experimental site information. (A) Experimental location; (B) experimental field; (C) cotton plant of the flowering stage for the first trial; (D) cotton plant of the early boll development stage for the second trial. The source of (A and B) were from https://earth.google.com/; (C and D) were taken by the corresponding author Hongyan Hu.

The field experiments were carried out in the trial base farm of the Institute of Cotton Research, Chinese Academy of Agricultural Sciences, Anyang, Henan Province, China (36° 05′10″N, 114° 30″60″ E) on 14 July 2020 (the first trial) and 22 August 2020 (the second trial), respectively (Figs. 1A and 1B). The cotton variety CCRI 79 at the flowering stage (Fig. 1C) with a Leaf Area Index (LAI) of 1.8 and the early boll development stage (Fig. 1D) with a LAI of 3.2 was adopted as the sampling plants in this work. The plant heights of the first and the second trials were 80–88 cm and 100–110 cm, respectively. The planting density and the row spacing of the field trial were 54,990 plants ha^−1^ and 80 cm, respectively.

### UAV spraying platform

**Figure 2:**
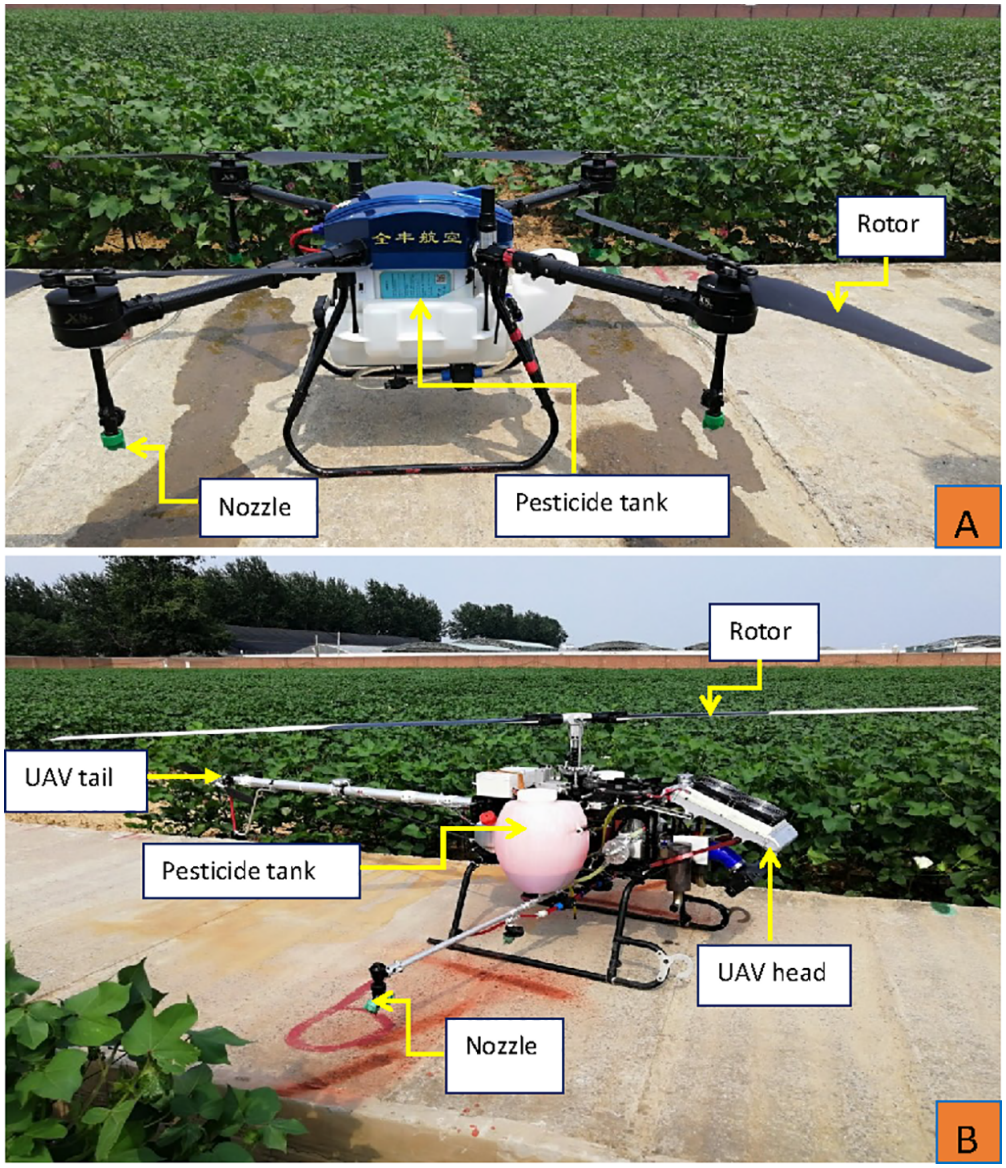
The four-rotor UAV 3WQFTX-10 (A) and the single-rotor UAV 3WQF120-12 (Version 2020) (B) used in the study. Photos were taken by the corresponding author Hongyan Hu.

In the first trial, an electric-powered UAV 3WQFTX-10 (Fig. 2A) and a petrol-powered single-rotor UAV 3WQF120-12 (Version 2020) (Fig. 2B) were used as the spraying platforms. Both UAVs were produced and provided by Anyang Quanfeng Aviation Plant Protection Technology Co., Ltd, China. In the second trial, only the single-rotor UAV was employed as the sprayer. The flight velocity of the two UAVs was 4 m s^−1^ in both trials based on the results of the previous study (Meng et al., 2019a). The main physical and operation parameters of the two UAVs can be found in the Supplemental File.

### Field trial design

**Figure 3:**
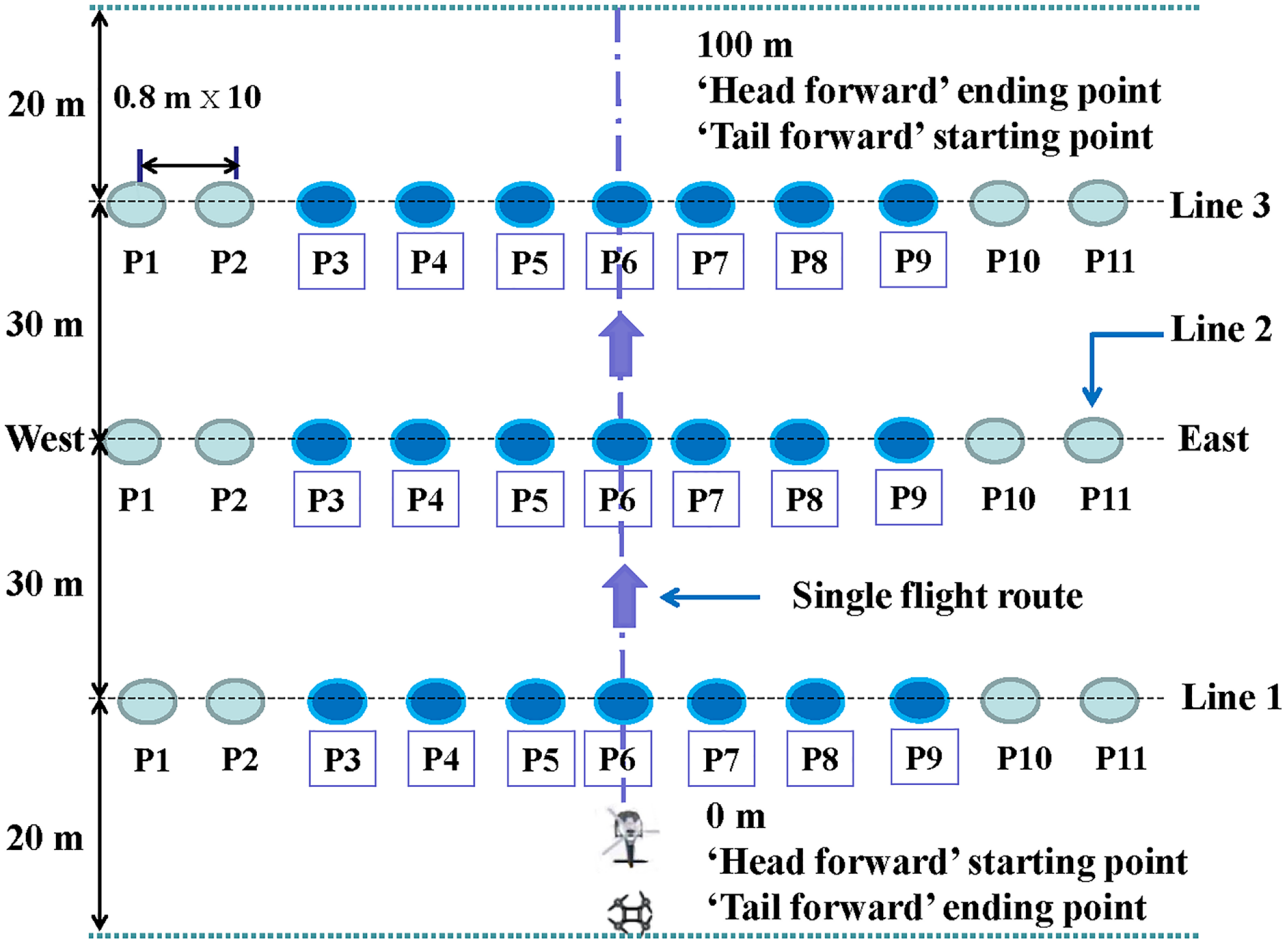
The layout of the field experiment.

In the first trial, we assessed the effect of flight heights and forward modes on the droplet distribution under a spraying volume of 12 L ha^−1^ for both UAVs, respectively. The flight heights of 1, 2, or 3 m s^−1^ and the forward modes of “head forward” or “tail forward” were paired for different treatments, respectively. The setup of six treatments for each UAV was presented in the tables shown in the Supplemental Files. For the forward modes of the single-rotor UAV, “head forward” meant that the UAV flew with its head forward while “tail forward” meant the opposite. For the forward modes of the four-rotor UAV, “head forward” indicated that the UAV took off from the site near the operator and then farther and farther from the operator when it was flying in the set flight route; “tail forward” referred to the opposite (Fig. 3). In the second trial, we evaluated the influence of the flight heights (1 and 2 m s^−1^) and spraying volumes (12.0 and 22.5 L ha^−1^) on the droplet distribution in the cotton canopy based on the results of the first trial.

A cotton field with a size of 20 m × 100 m was designed for the evaluation experiment. The layout of the sampling points was shown in Fig. 3. Three sampling lines with an interval of 30 m were perpendicular to the UAV flight route. In each sampling line, eleven plants (referred to as P1 to P11, respectively) were used for placing water sensitive papers (WSPs). The interval between rows was 80 cm. According to the row spacing, the point of P6 was regarded as position 0, while these of P1 to P5 and P7 to P12 were considered as positions −4, −3.2, −2.4, −1.6, −0.8, and 0.8, 1.6, 2.4, 3.2, 4, respectively. In the first trial, the WSPs were arranged on 27 leaves (referred to as L1–L27) from the top to the bottom canopy. In the first trial, leaves L1 to L9 were classified as the upper layer, while leaves L10 to L18 were classified as the middle layer and the remaining nine leaves (L19–L27) were all counted as the bottom layer. In the second trial, only seven plants (P3 to P9) were adopted to collect droplets in each sampling line based on the droplet distribution results of the first trial. In the second trial, the cotton canopy was divided into eight directions, *i.e*., East, Southeast, South, Southwest, West, Northwest, North, and Northeast, respectively. For each direction, two WSPs were placed on two leaves in the middle layer and one WSP was placed on one leaf in the bottom and the upper layers, respectively. Treatment information was summarized in two tables, respectively (please refer to Supplemental Files).

### Droplet deposition evaluation

Droplet deposition (μL cm^−2^) was chosen as the metric to assess the droplet distribution in the cotton canopy under different treatments. Droplets were collected by WSPs first in the cotton field and then analyzed by a scanner-based image processing software DepostiScan™ (USDA, Wooster, OH, USA) (Hoffmann & Hewitt, 2005; Zhu, Salyani & Fox, 2011) to obtain the droplet deposition data. The mean and standard error (SE) of the three layers in each treatment in both trials were calculated, respectively.

### Meteorological condition

The air temperature, wind direction, and wind speed were measured by a Kestrel 5500 Link micro weather station (Kestrel Company, Decatur, IL, USA) every 2 s on the field trial day. The weather station was placed in a position around 20 m from the UAV taking-off site and approximately 1.5 m above the upper canopy of the cotton plants. All treatments of the spraying experiments were conducted under a wind speed condition of less than 3 m s^−1^ to avoid droplet drift. Data on the air temperatures, wind directions, and wind speeds can be found in the Supplemental File.

### Statistical Analyses

The means and SE were performed by SPSS Statistics 24.0 (IBM Co, Armonk, NY, USA) software. Means of the droplet deposition were explicitly expressed as bar charts drawn by Origin 2019 (Academic) (Origin Lab, Northampton, MA, USA). The droplet deposition (μL cm^−2^) on 27 leaves of the eleven sampling plants in the first trial was shown using figures drawn by the Helm software.

## Results

### Droplet distribution of the first field trial

#### Droplet distribution of the 11 sampling cotton plants

**Figure 4:**
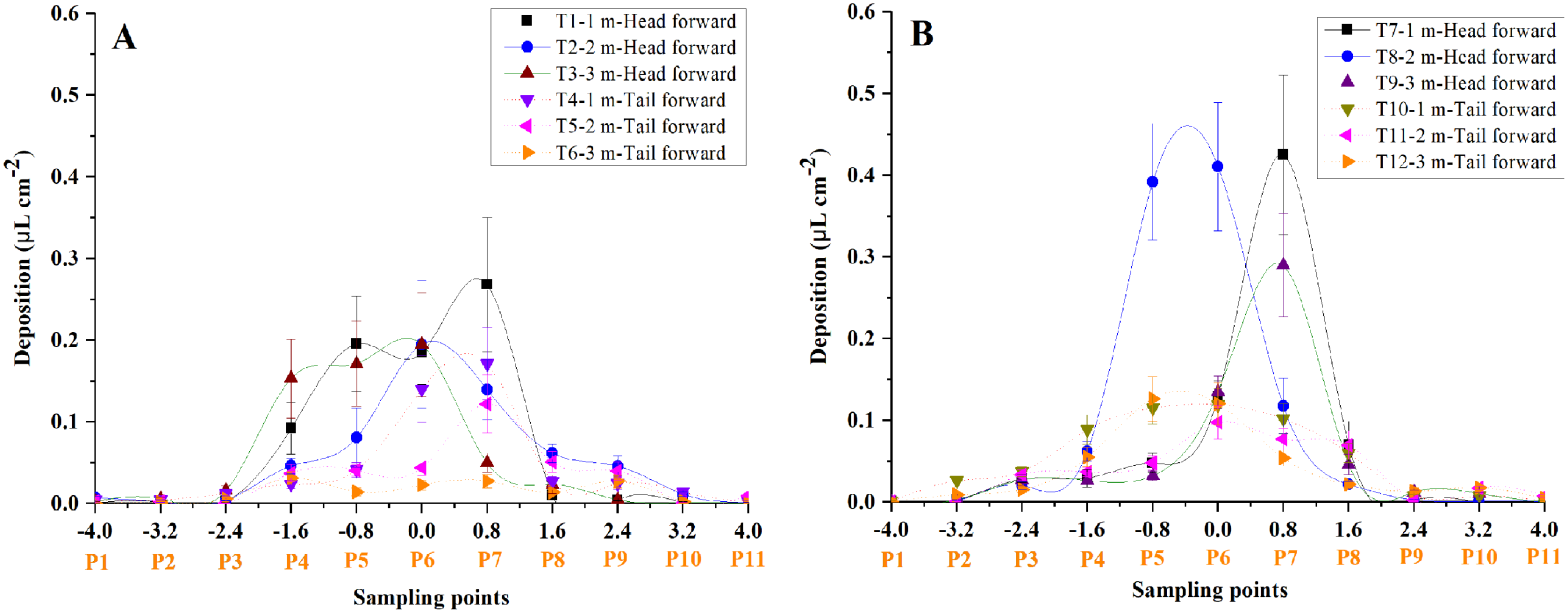
The droplet distribution in the sampling line of the single-rotor (A) and the four-rotor (B) UAVs in the first field trial.

The average droplet deposition of the whole cotton canopy at different sampling points was illustrated in Fig. 4. Most of the droplets were observed to deposit within the sampling points −1.6 and 1.6. In most cases, the total droplet deposition of the “head forward” treatments was higher than that of the “tail forward” treatments for both UAVs under the three flight heights, respectively. Furthermore, the total droplet deposition of the four-rotor UAV (Fig. 4B) outperformed the single-rotor UAV (Fig. 4A) under the three flight heights, respectively.

**Figure 5:**
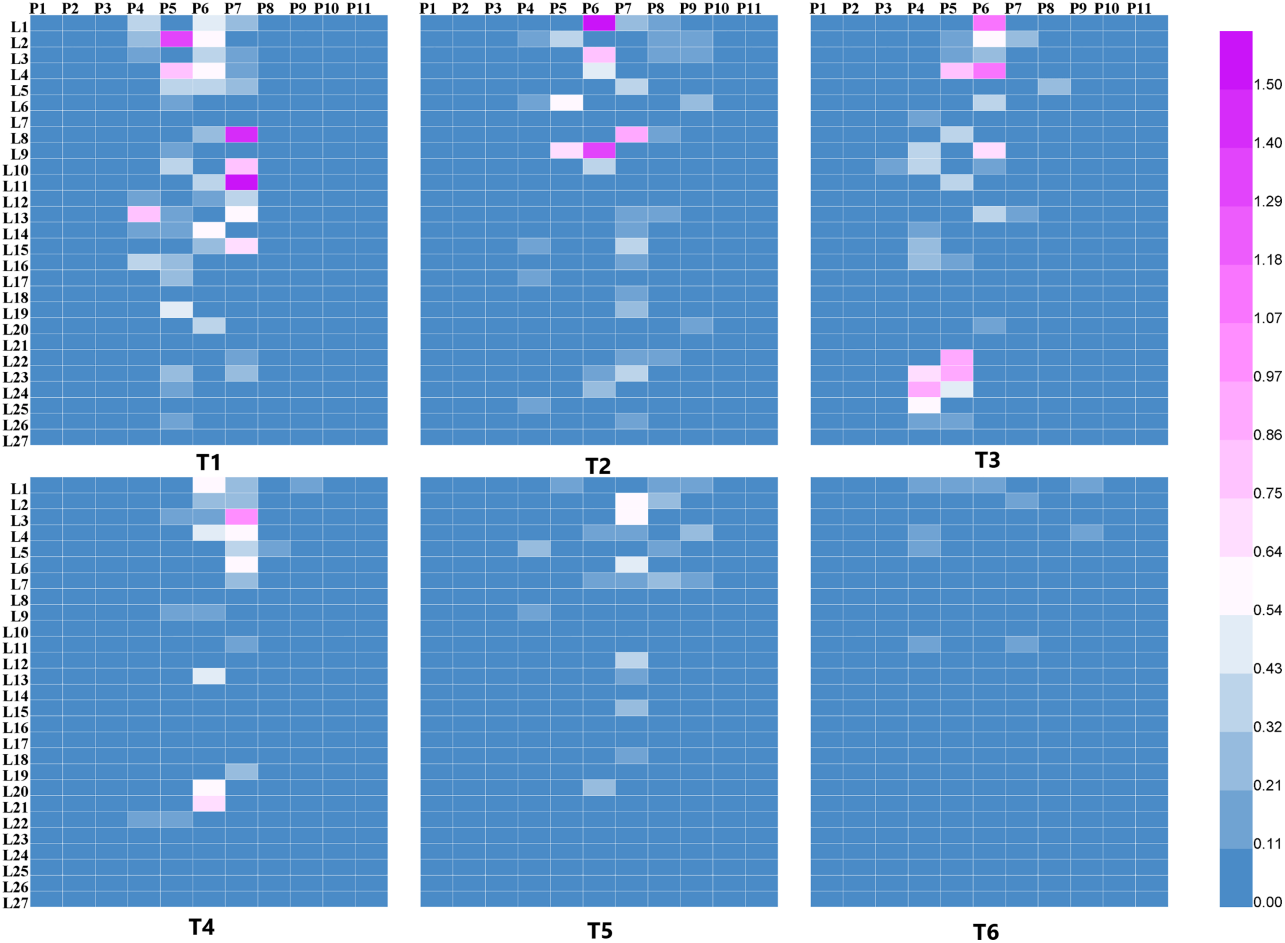
The droplet deposition (μL cm^−2^) on 27 leaves in the 11 sampling plants using the single-rotor UAV.

**Figure 6:**
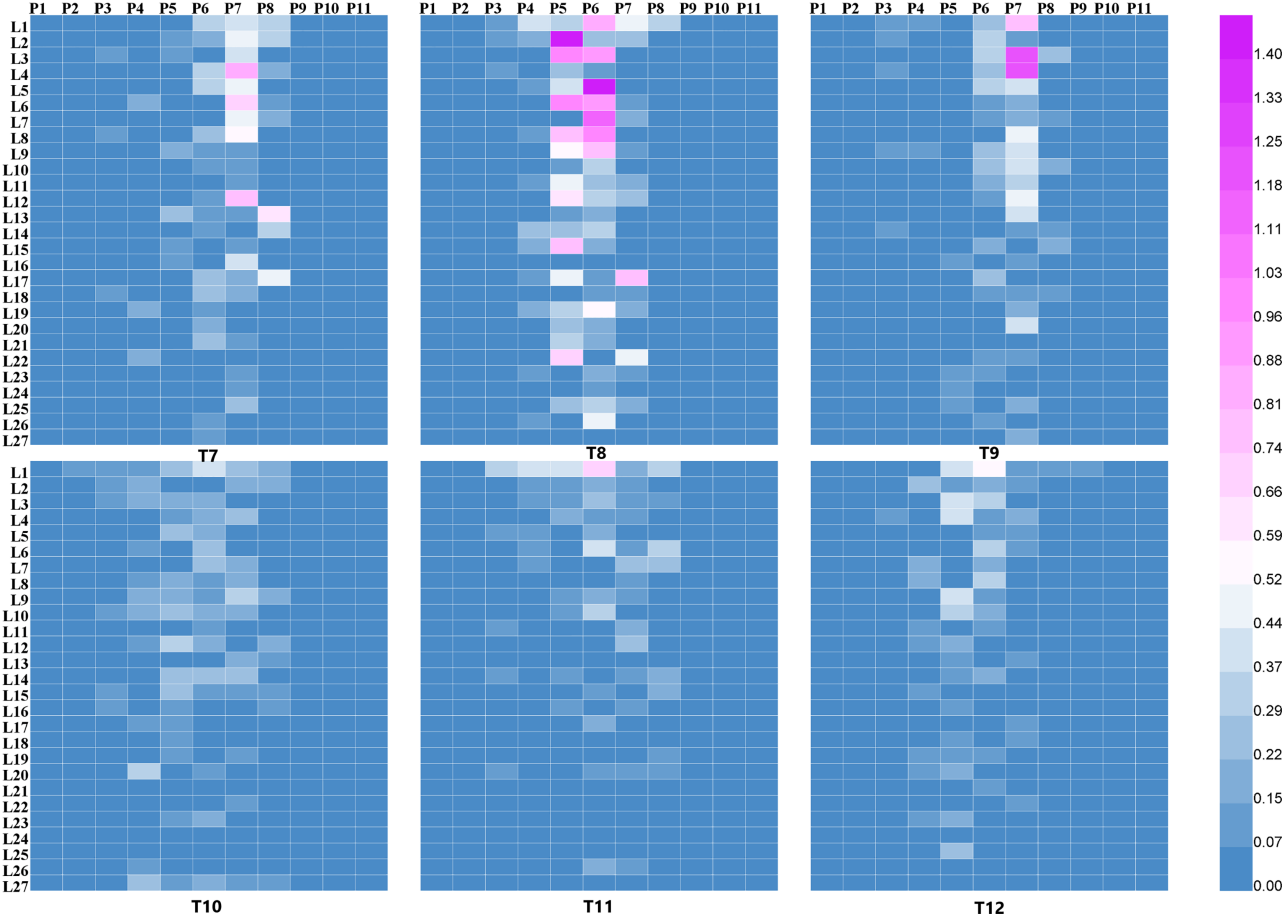
The droplet deposition (μL cm^−2^) on 27 leaves in the 11 sampling plants using the four-rotor UAV.

The droplet deposition of 27 leaves in the 11 sampling plants was shown in Figs. 5 and 6. The droplet deposition of 27 leaves in each cotton plant under different flight heights (1, 2, and 3 m) and forward modes (“head forward” and “tail forward”) were analyzed in the following section.

As illustrated in Fig. 5, for the single-rotor UAV treatments (T1 to T6), most of the droplets were deposited on plants P4 to P8, very few droplets landed on plants P3 and P9, and almost no droplets were collected from plants P1, P2, P10, and P11. Compared with “tail forward” (T4, T5, and T6), more droplets were collected while the UAV flew with the “head forward” (T1, T2, and T3) under the same flight height. These droplet distribution characteristics were consistent with Fig. 4A. When the UAV flew at 1 m above the cotton canopy, droplet distribution of the “head forward” treatment (T1) was more even than that of the corresponding “tail forward” treatment on plants P4 to P7 at the same leaf position. For the group with a flight height of 2 m (T2 and T4), droplet deposition of leaves in plants P4 to P9 in both forward modes was similar except for that of L1 and L9 of plant P6 in T2. For the group with a flight height of 3 m (T3 and T6), the “head forward” treatment (T3) obtained more droplets than that of the “tail forward” treatment (T6) at the same leaf position. As shown in Fig. 6, when the four-rotor UAV was adopted as the sprayer, droplets were mainly deposited on plants P4 to P8, and some of the droplets deposited on the top leaves of plants P3 and P9 occasionally for both forward modes. Droplet deposition of the paired treatments with “head forward” and “tail forward” (T7 and T10, T9 and T12) was similar under the same flight height (1 and 3 m) and similar wind conditions. However, under different wind conditions, droplet deposition of the group with a flight height of 2 m showed different distribution in the cotton canopy, *i.e*., the droplet deposition of the “head forward” treatment (T8) was higher than that of the “tail forward” treatment (T11) for most of the sampling leaves at the same leaf position. Therefore, under the same flight height and wind condition, droplet distribution of the “head forward” and “tail forward” treatments were identical for the four-rotor UAV and that of the “head forward” outperformed the “tail forward” for the single-rotor UAV, based on the droplet deposition values of the sampling leaves.

#### Droplet distribution in the three layers of the sampling plants P3 to P9

The results described above indicated that the droplets deposited primarily on plants P4 to P8 and occasionally deposited on plants P3 and P9. Figures 5 and 6 also showed that the droplet deposition of different leaves varied dramatically. For further understanding of the droplet distribution in the cotton canopy, 27 leaves of each sampling plant were categorized into the three group layers as mentioned in the Materials and Methods section. Droplet deposition values of the three layers were plotted using a Histogram with an error.

**Figure 7:**
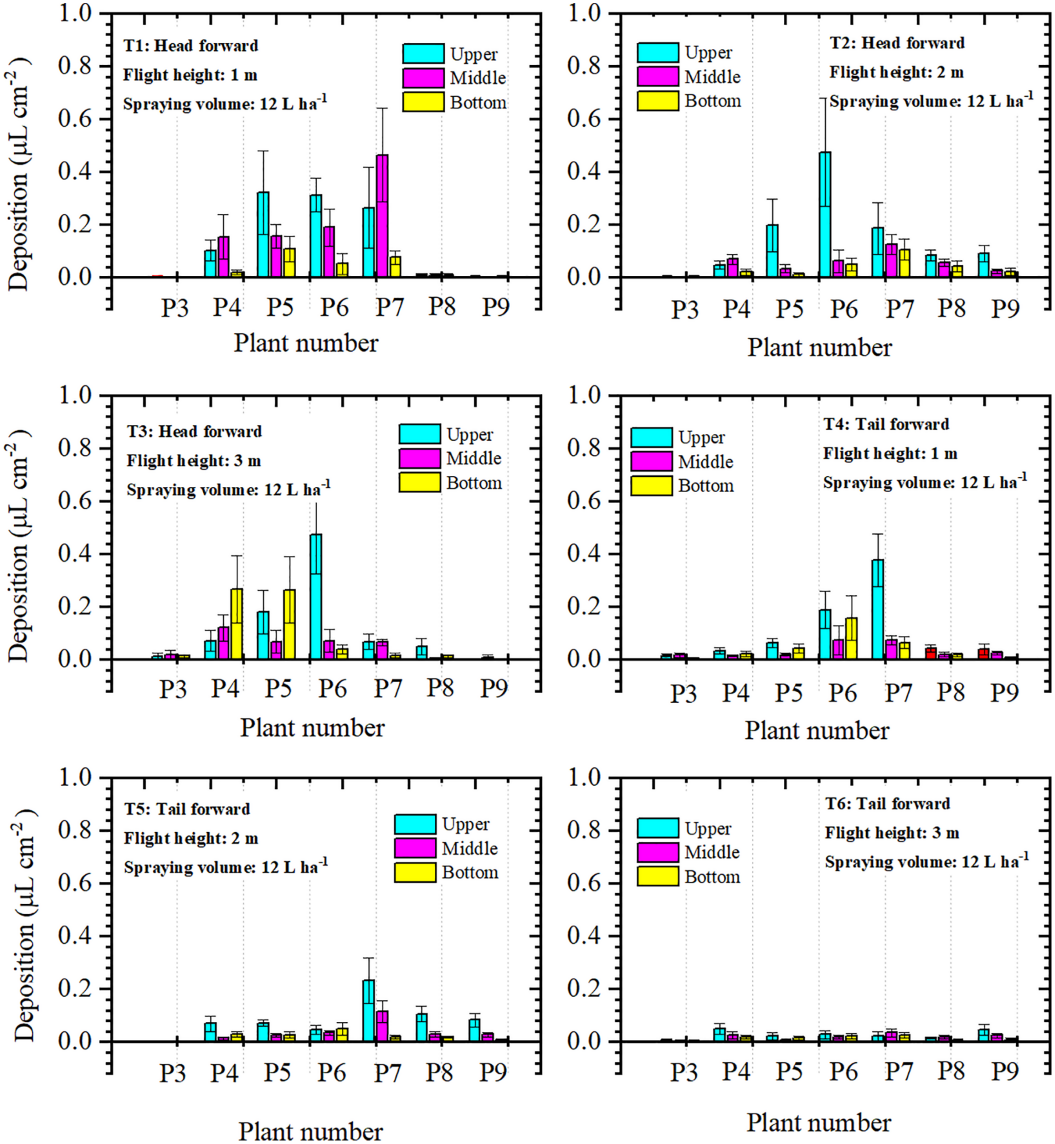
The droplet deposition (μL cm^−2^) of the upper, middle, and bottom layers of the single-rotor UAV.

Droplet deposition of the single-rotor UAV treatments in the three layers was illustrated in Fig. 7. The values of droplet deposition differed greatly between the treatments and the three layers within a treatment. For “head forward” treatments, droplet deposition of the upper layer was higher than that of the middle and bottom layers in most cotton canopies. However, for some cotton plants in T1 and T3, droplet deposition of the middle or bottom layer was higher than that of the upper layer (P4 and P7 in T1; P4, P5, and P7 in T3). For the “tail forward” treatments, droplet deposition of the three layers was relatively lower than that of the corresponding “head forward” treatment and the upper layer obtained more droplets in most cases. Droplet deposition of the three layers in the treatment with a flight height of 3 m (T6) was relatively less but more even than that of the treatment with a flight height of 2 m (T5) and 1 m (T4). For the single-rotor UAV, from the aspect of droplet deposition of the three layers, the upper layer accessed more droplets in most cases for both forward modes and the “head forward” outperformed the “tail forward”.

**Figure 8:**
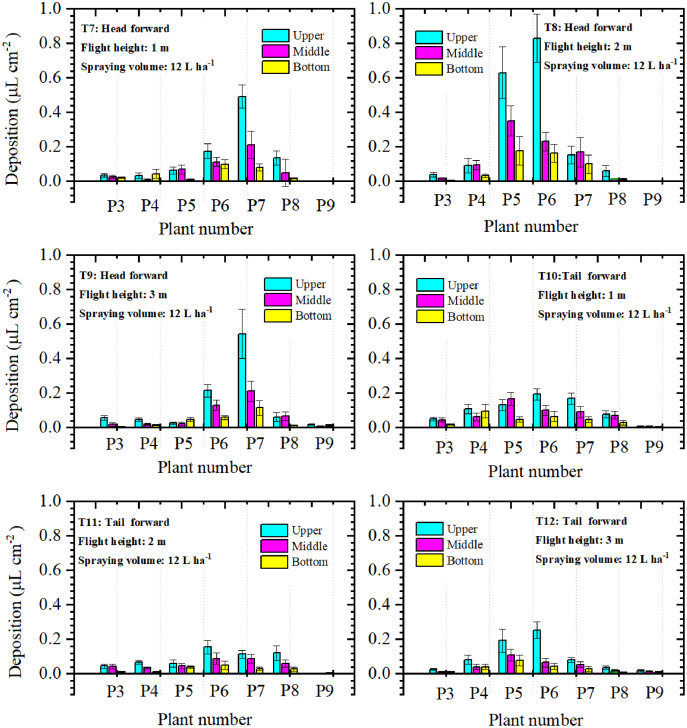
The droplet deposition ( μL cm^−2^ ) of the upper, middle, and bottom layers of the four-rotor UAV.

Droplet deposition of the four-rotor UAV treatments in the three layers was shown in Fig. 8. During both forward modes, the droplet deposition value of the upper layer was the highest in most sampling plants. For the “head forward” treatments, droplet deposition of the upper layers of P7 in T7 and T9, P5, and P6 in T8 was significantly higher than that of the other sampling plants. For the other sampling plants, droplet deposition in the three layers was relatively close within the same plant. For “tail forward” treatments, droplet deposition of the upper canopy was relatively stable and that of the middle and bottom layers was almost the same. Comparatively, the middle and bottom layers of the four-rotor UAV treatments accessed much fewer droplets than that of the upper layer for most sampling plants. For four-rotor UAV, from the aspect of droplet deposition of the three layers, the upper layer also accessed most of the droplets but that of the middle and bottom layers was much lower than that of the corresponding treatments of the single-rotor UAV.

For further analysis of the droplet distribution in different layers of the cotton canopy, the percentages of droplet deposition in the three layers were compared in Table 1. The total droplet deposition of the “head forward” treatments (T1, T2, and T3) was around 1.7–4.3 times that of the “tail forward” treatments (T4, T5, and T6) under the same flight height. For the single-rotor UAV, droplet deposition percentages of the upper layer in “head forward” treatments were 44.95% (T1), 63.15% (T2), and 46.68% (T3), respectively. Some of these values were lower than that of the corresponding “tail forward” treatments (T1 and T4), some were the opposite (T2 and T4), and some were almost the same (T3 and T6). For four-rotor UAV treatments, the total droplet deposition of “head forward” treatments was larger than that of the corresponding “tail forward” treatments. The percentages of droplet deposition in the upper layers were between 50.37% to 56.57% except for T10 (46.42%) and these in the middle layers were between 25.47% to 34.09%. The “tail-forward” treatment with a flight height of 1 m had the most outstanding performance in droplet penetration among the six four-rotor UAV treatments and the “head forward” treatment with a flight height of 2 m had the poorest droplet penetration among the six single-rotor UAV in the first filed trial.

**Table 1:** The droplet deposition percentages in the cotton canopy of the first trial.

	Total droplet Deposition (μL cm^−2^)	Percentages (%) of droplet deposition in three layers		
Treatments		Upper	Middle	Bottom
T1	2.276 ± 0.134	44.95	43.01	12.04
T2	1.723 ± 0.105	63.15	21.76	15.09
T3	1.838 ± 0.119	46.68	19.70	33.62
T4	1.352 ± 0.086	57.51	18.42	24.08
T5	1.01 ± 0.054	60.40	24.65	14.95
T6	0.432 ± 0.012	45.14	31.02	23.84
T7	2.178 ± 0.171	50.37	32.74	16.90
T8	3.168 ± 0.215	56.72	27.72	15.56
T9	1.706 ± 0.122	56.57	27.90	15.53
T10	1.590 ± 0.054	46.42	34.09	19.50
T11	1.098 ± 0.042	51.28	33.06	15.66
T12	1.221 ± 0.062	56.51	25.47	18.02

### Droplet distribution of the second field trial

The results of the first trial indicated that forward mode was not a key factor in the droplet distribution for the four-rotor UAV under the same wind condition and flight height. Thus, only the single-rotor UAV was adopted as the sprayer in the second field trial. The results of the first trial also indicated that the droplet deposition of all treatments with a spraying volume of 12 L ha^−1^ was relatively few. Thus, the spraying volume of 22.5 L ha^−1^, which was the spraying volume recommended for defoliation application (Meng et al., 2019a), was used as another spraying volume level in the second field trial. In addition, the total droplet deposition of the treatment with a flight height of 3 m was only around 19–43% of the other treatments in the first trial. Thus, only the flight heights of 1 and 2 m were adopted in the second field trial. Droplet deposition in the eight directions of the canopy was evaluated in the second trial for further understanding of droplet distribution in the cotton canopy.

#### Droplet distribution in the three layers of the sampling plants

**Figure 9:**
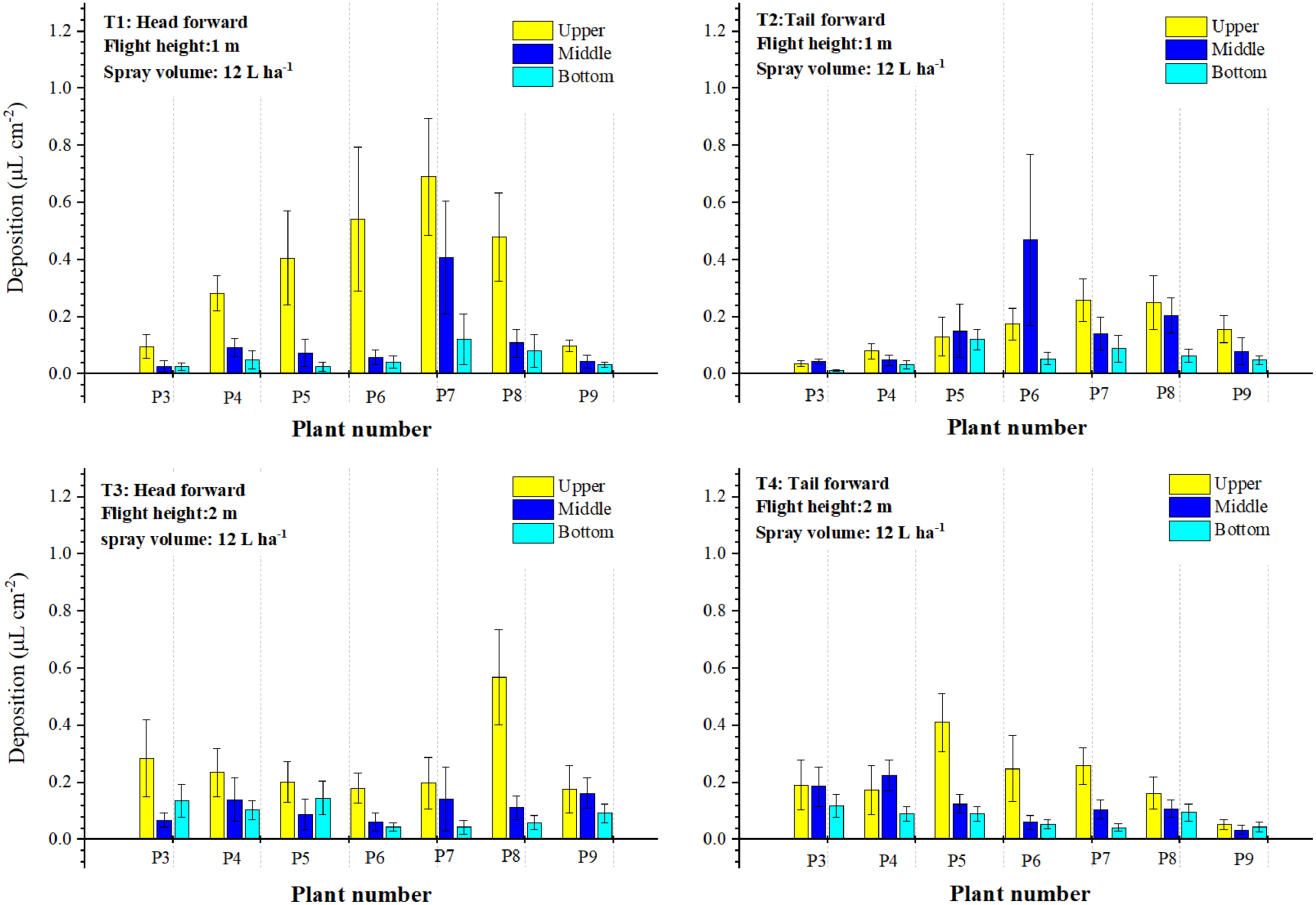
The droplet deposition (μL cm^−2^) of the treatments with a spraying volume of 12.0 L ha^−1^ in the three layers.

Droplet depositions in the three layers of the treatments with a spraying volume of 12.0 L ha^−1^ were shown in Fig. 9 and the percentages of droplets deposition in the three layers were presented in Table 2. For treatments with a flight height of 1 m, the “head forward” (T1) facilitated more droplets to deposit on the upper layer of plants P4 to P8 than that of the “tail forward” (T2). Droplet deposition values of the upper layer of T1 were all higher than these of the bottom and middle layers. However, the same phenomenon was not observed in T2. The upper layer of the three sampling plants (P3, P5, P6) of T2 accessed fewer droplets than that of the middle layer. Furthermore, the droplet deposition value of the middle layer of P6 was the highest in T2. The upper layer of T1 obtained 68.41% of the droplets, while that of the middle and bottom layers was 21.52% and 10.06%, respectively. In T2, droplet deposition percentages of the upper, middle, and bottom layers were 41.05%, 43.00%, and 15.95%, respectively. When the flight height increased to 2 m, droplet distribution was more even than that of the 1 m. For the “head forward” treatment (T3), droplet deposition values of the upper layer were all higher than that of the bottom and middle layers. The highest value was observed in the upper layer of P8 in T3. For the “tail forward” treatment (T4), the upper layer of P5 obtained the highest droplet deposition. About 56.85% of the droplets were deposited on the upper layer in T3, while that of T4 was 52.10%; the percentage of droplet deposition in the middle layer of T3 was 5.55% less than that of T4 (29.39%). However, the proportion of droplets in the bottom layers of T3 (19.31%) and T4 (18.50%) was almost the same. Thus, droplet deposition of “head forward” was higher than that of “tail forward” for the treatment with a spraying volume of 12 L ha^−1^ and a flight height of 1 or 2 m. However, the “tail forward” treatment facilitated more droplets to deposit in the lower part of the cotton canopy (middle and bottom layers).

**Table 2:** The droplet deposition percentages in the cotton canopy of the second trial.

	Total droplet Deposition (μL cm^−2^)	Percentages (%) of droplet deposition in three layers		
Treatments		Upper	Middle	Bottom
T1	3.796 ± 0.538	68.41	21.52	10.06
T2	2.658 ± 0.106	41.05	43.00	15.95
T3	3.242 ± 0.115	56.85	23.84	19.31
T4	2.875 ± 0.093	52.10	29.39	18.50
T5	6.671 ± 0.248	57.11	27.67	15.22
T6	5.938 ± 0.162	48.43	29.91	21.66
T7	6.696 ± 0.160	49.45	29.58	20.97
T8	7.695 ± 0.219	44.50	31.42	24.08

**Figure 10:**
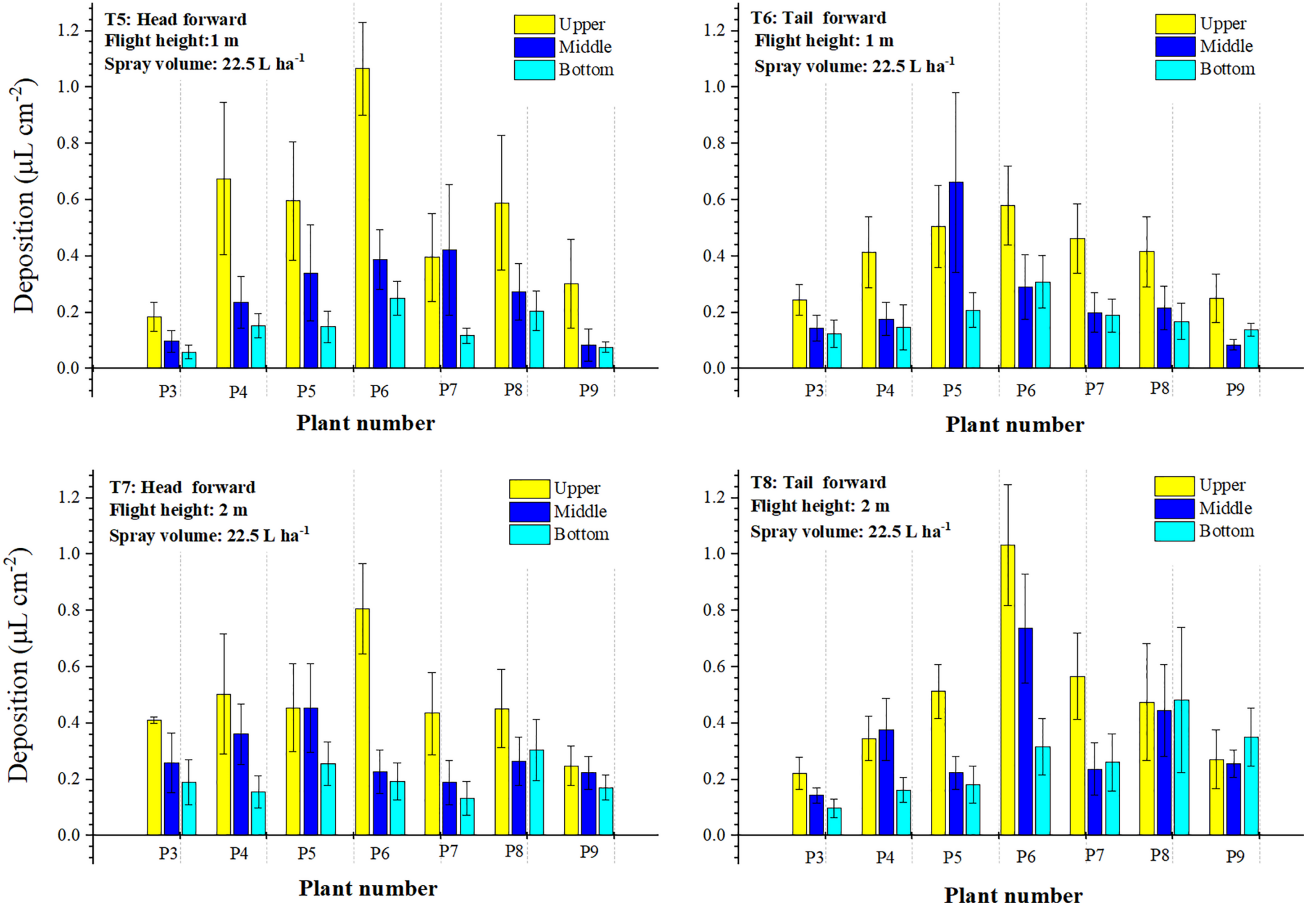
The droplet deposition (μL cm^−2^) of the treatments with a spraying volume of 22.5 L ha^−1^ in the three layers.

**Figure 11:**
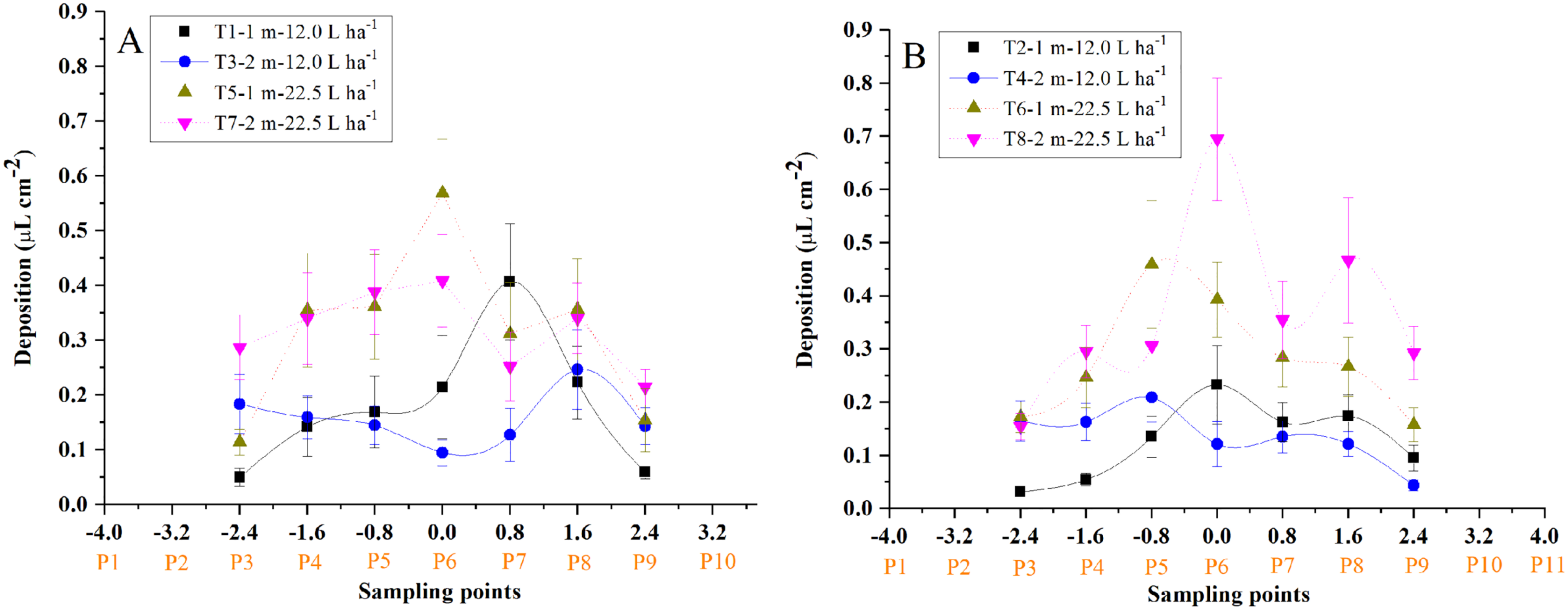
The droplet distribution of the single-rotor UAV under “head forward” (A) and “tail forward” (B) flight modes with different spraying volumes in the second field trial.

Droplet depositions in the three layers of the treatments with a spraying volume of 22.5 L ha^−1^ were shown in Fig. 10 and the percentages of droplet deposition in the three layers were presented in Table 2. The increase of the spraying volume from 12.0 to 22.5 L ha^−1^ significantly improved the droplet distribution of the sampling plants. The improvement could be observed in both the uniformity and droplet deposition. In T5, all sampling plants in the upper layer had droplet deposition of 0.302 to 0.675 μL cm^−2^ except the highest value in P6 (1.066 μL cm^−2^) and the lowest value in P3 (0.185 μL cm^−2^). Droplet deposition of P7 in the middle layer (0.432 μL cm^−2^) was slightly higher than that of the upper layer (0.396 μL cm^−2^). The bottom layer of T5 obtained less droplet deposition than that of the upper and middle layers. The total droplet deposition of T5 was 6.671 μL cm^−2^, which was nearly twice that of the treatment with the same forward mode and height (T2). In T5, around 57.11% of the droplet distributed in the upper layer, and about 27.67% and 15.22% of the droplets were deposited in the middle and bottom layers, respectively. In T6, droplet deposition of plants at both ends of the sampling line (P3 and P9) was between 0.245–0.251 μL cm^−2^. A small fluctuation was observed in the droplet deposition values of P4 to P8, which ranged from 0.415–0.580 μL cm^−2^. In the middle layer, the fluctuation of the droplet deposition was dramatic, which presented that the highest point was 0.662 μL cm^−2^ and the lowest point was only 0.085 μL cm^−2^, and the values of the others in the range of 0.146–0.290 μL cm^−2^. However, droplets in the bottom layer deposited relatively uniformly except P6 with the highest value of 0.309 μL cm^−2^. The total droplet deposition of T6 was 5.938 μL cm^−2^, which was around 0.70 μL cm^−2^ less than that of T5. The percentages of droplet deposition in the three layers were 48.43% (upper), 29.91% (middle), and 21.66% (bottom), respectively. When the flight height increased to 2 m, droplet deposition in the upper layer had a small fluctuation except for the highest point of P6 (0.806 μL cm^−2^) and the lowest point of P9 (0.249 μL cm^−2^) in T7. The total droplet deposition of 6.696 μL cm^−2^ was collected, and around 49.45%, 29.58%, and 20.97% of the droplets were distributed in the upper, middle, and bottom layers, respectively. For the “tail forward” treatment (T8), droplet deposition in each layer had a large fluctuation. However, the total droplet deposition of this treatment was about 1.0 μL cm^−2^ higher than that of T7. Around 44.50% of the droplets were deposited in the upper layer, and about 31.42% and 24.08% of the droplets were deposited in the middle and bottom layers, respectively. Figure 11 further showed the influence of the flight height and spraying volume on the droplet distribution. During both forward modes, the total droplet depositions increased with the increase of the spraying volume under the same flight height. Droplet distribution uniformity of the treatment with a flight height of 2 m was better than that of the 1 m under the same spraying volume.

According to the pooled results of the second trial, it could be concluded that the treatment with a spraying volume of 22.5 L ha^−1^ and the “head forward” could obtain more droplets in the upper part of the canopy, and that of “tail forward” facilitated more droplets to deposit in the lower part of the canopy.

#### Droplet deposition in the eight directions

**Figure 12:**
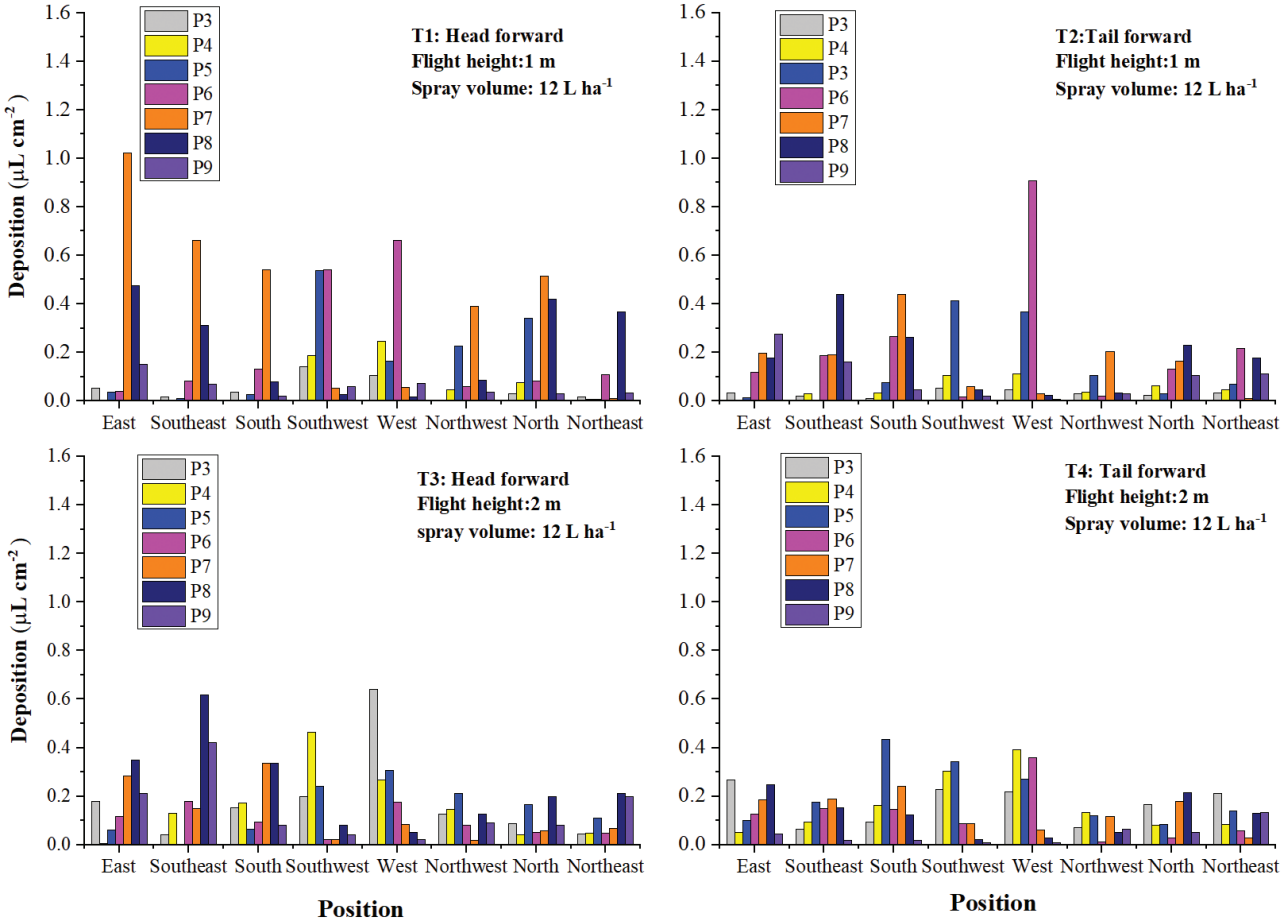
The droplet deposition (μL cm^−2^) of the treatments with a spraying volume of 12.0 L ha^−1^ in the eight directions.

**Figure 13:**
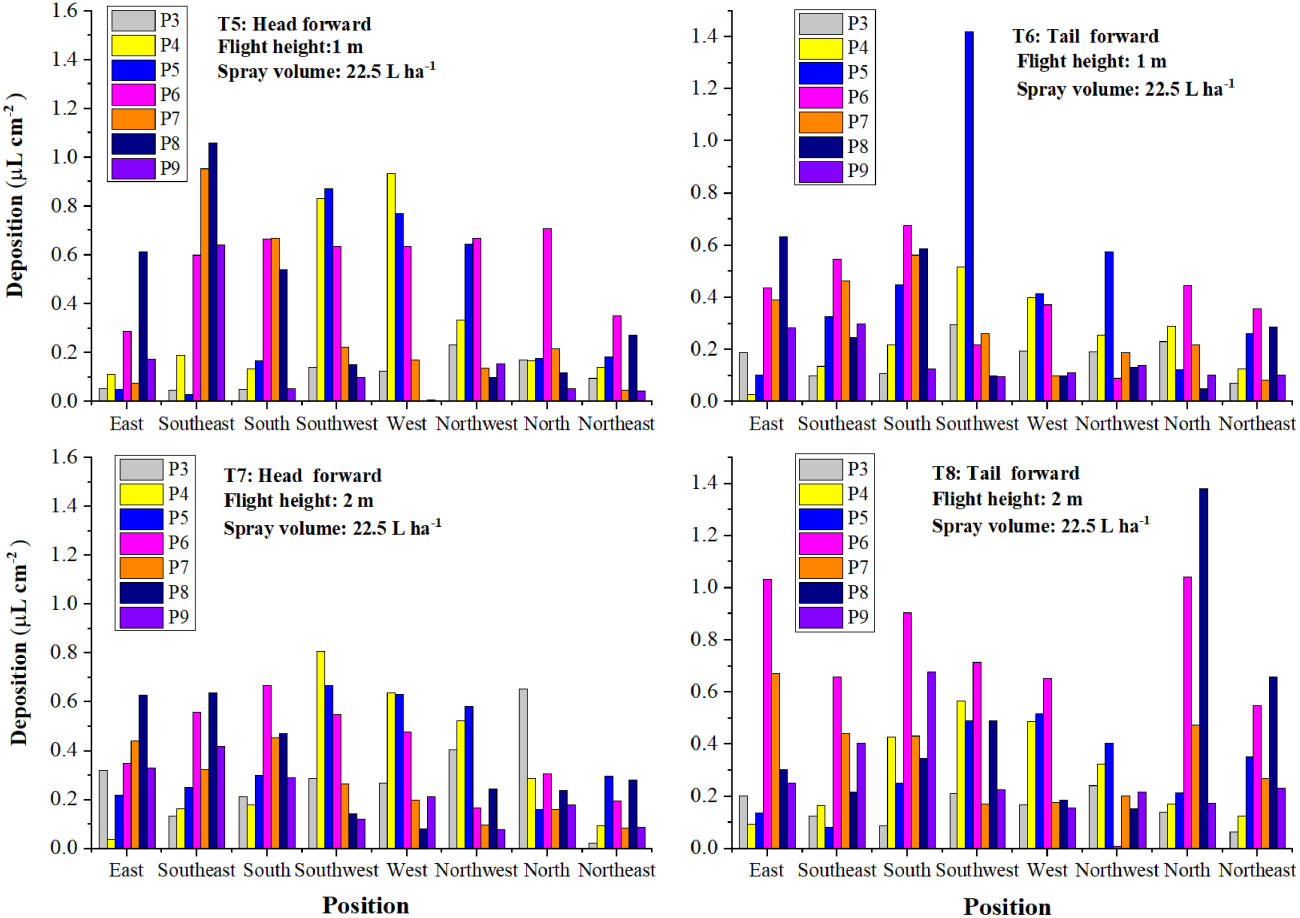
The droplet deposition (μL cm^−2^) of the treatments with a spraying volume of 22.5 L ha^−1^ in the eight directions.

From the analysis of the results above, it could be seen that droplet deposition of the three layers in the same treatment varied remarkably. For further investigation of the droplet distribution in the cotton canopy, droplet distribution in the eight directions was shown in Figs. 12 and 13.

As illustrated in Fig. 12, the droplet deposition of two or more plants was higher than 0.2 μL cm^−2^ in all directions except the South and Northeast directions in T1. Droplet deposition of most plants in the three directions, Northwest, North, and Northeast, was less than 0.2 μL cm^−2^ in T2, T3, and T4. Droplets were mainly deposited in the remaining six directions. It could be due to the wind direction, wind speed, and the downwash generated by rotors. As shown in Fig. 13, the increase in spraying volume made droplet deposition in the eight directions more even than that of the corresponding treatments with a spraying volume of 12 L ha^−1^. The droplet distribution uniformity of the treatment with a spraying volume of 22.5 L ha^−1^ and a flight height of 2 m (T7) outperformed that of the other three treatments (T5, T6, and T8).

## Discussion

The effect of the flight heights, forward modes, and spraying volumes on the droplet distribution of UAV spraying in the cotton canopy was evaluated in this study. Those factors had different impacts on the droplet distribution. Droplet distributions of the whole canopy were variable remarkably between treatments due to the differences in application parameters. In addition to the difference between treatments, droplet deposition in the three layers and eight directions within a treatment also varied significantly. These variabilities were also verified from the droplet distribution results of the eight directions in the same canopy under the same application parameters.

Droplet distribution in the crop canopy was affected notably by the flight height. More droplets drifted away when a UAV flew at a relatively high altitude (Xue et al., 2014; Lou et al., 2018). The flight heights of the single-rotor UAV combined with a “head forward” or “tail forward” forward mode had a different effect on the droplet distribution. In the first trial, the “head forward” treatment with a flight height of 1 m obtained the most droplet deposition in total, while that of 2 and 3 m were close to each other. However, the total droplet deposition of “tail forward” treatment declined with the increase of the flight height. For most “tail forward” treatments, the droplets depositing on the upper layers were more than those on the middle and bottom layers but less than those of the corresponding “head forward” treatments. Previous research conducted in the wheat field reported that “head forward” was more suitable for droplets to deposit on the targets when a single-rotor UAV flew at the parameters combined a flight height of 3 m and a flight speed of 5 m s^−1^ under wind speed of 1.2 m s^−1^ (Wang et al., 2016). The result shown in Fig. 6 was inconsistent with the previous research (Wang et al., 2016). Besides the difference in the flight heights, it may be contributed to the canopy structure and rotor size of the single-rotor UAV. Canopy structure was a noticeable factor impacting droplet distribution in the crop canopy. The rotor diameter of the single-rotor UAV used in the previous study was 1,800 mm (Wang et al., 2016) and that of this study was 3,400 mm. The UAV rotor size correlated with the strength and distribution of the downwash, which had a significant effect on the droplet distribution in the crop canopy. For four-rotor UAV, the “head forward” treatment with a flight height of 2 m obtained the most droplets in the six treatments, while that of the corresponding “tail forward” treatment accessed the least droplets. It might be caused by the weather condition during the spraying implementation as mentioned in the ‘Results’ section. Under a similar wind condition, the droplet distribution of the four-rotor UAV in the three layers of the cotton canopy was not affected significantly by the flight heights and forward modes in this study. It might be caused by the downwash distribution of the UAV rotors. For four-rotor UAV, the downwash distribution on the cotton canopy might be similar at the flight height with a range from 1 to 3 m, which resulted in similar droplet distribution in the cotton canopy.

Droplet deposition in the crop canopy could be variable significantly at different application spraying volumes (Giles & Billing, 2015; Meng et al., 2019a). Under the same flight height and speed, the increase of the spraying volume from 12 to 22.5 L ha^−1^ remarkably increased droplet deposition in the second trial for both “head forward” and “tail forward” treatments using the single-rotor UAV. Droplet depositions increased in the three layers with the increase of the spraying volume. Moreover, the increase of droplet deposition in the upper layer was the most noticeable in the three layers. It may be due to no obstacles between the nozzles and the upper canopy. With the same flight height and spraying volume, compared to “head forward”, “tail forward” facilitated more droplets to deposit in the lower canopy, as mentioned above.

In addition to the flight height and spraying volume, UAV downwash generated by rotors and UAV structure also had a remarkable effect on the droplet distribution. Droplet deposition zone and distribution were both affected by the UAV downwash significantly due to the contact area of downwash and crop canopy determining droplet deposition location (Tang et al., 2017; Wu et al., 2019). The downwash distribution of aircraft was affected by the structure of the spraying platform and rotor size (Li et al., 2019). From the results of the first trial, it was observed that the single-rotor and four-rotor UAVs had a different effect on the droplet distribution. Under the same flight height, droplet penetration of the single-rotor UAV was differentiated from that of the four-rotor UAV in the three layers. In addition, the forward modes of the single-rotor UAV had a remarkable effect on the droplet distribution in a single spraying swath, which was consistent with the previous study (Wang et al., 2016). The asymmetric structure between the head and tail of a single-rotor UAV may result in different downwash distribution in the “head forward” and “tail forward” treatment. Four-rotor UAV has the structure of bilateral symmetry and anteroposterior symmetry. In theory, both “head forward” and “tail forward” forward modes have similar droplet distribution under the same flight parameters and weather conditions. However, the droplet distribution of the corresponding groups (T8 and T11, T9 and T12) in the first trial showed different characteristics, which may contribute to the weather condition, such as wind speed fluctuations and wind direction (Nuyttens et al., 2006). Under the same flight height, the higher wind speed was more likely to result in more drift in the field condition. Both T8 and T11 had the same wind direction but different wind speeds (data shown in the Supplemental Files). Total droplet deposition of T8 was almost twice as big as T11. The high wind speed might result in the low droplet deposition of T11. Compared to T11, the wind speed of T8 was more suitable for droplets to deposit on leaves. Thus, droplet deposition values of T8 were higher than that of T11, of which, plants P5 and P6 accessed more droplets obviously. In addition to the factors mentioned above, the diversity of the cotton canopy characteristics might also contribute to the differences in the droplet distribution between treatments and canopy layers in both field trials. These potential influencing factors would be taken into consideration of investigating factors in future work for an in-depth analysis of droplet distribution in the cotton canopy.

## Conclusions

The following conclusions are supported by the results of this work:

1. Under the same flight height and similar wind speed, forward modes of the single-rotor UAV have a noticeable effect on the droplet distribution in the cotton canopy but those of the four-rotor UAV have no significant effect on the droplet distribution.
2. The flight height and spraying volume have significant impacts on the droplet distribution of UAV spraying in the cotton canopy.
3. Droplet deposition of different leaves, canopy layers and directions vary significantly under the same application parameters, which indicate the poor uniformity of droplet distribution of UAV spraying in the cotton canopy.
4. For the single-rotor UAV spraying, a flight height of 2 m combined a spraying volume of 22.5 L ha^−1^ is the optimal spraying parameters in this work. Under these spraying parameters, the “head forward” is a better alternative for harvest aid spraying and the “tail forward” is suitable for controlling targets of the lower part of the cotton canopy.
5. The upper layer droplet distribution of the four-rotor UAV outperforms the single-rotor UAV under the same flight height and spraying volume. Thus, the four rotor UAV might be a better potential option for spraying pesticides to control the targets of the upper canopy.

In all, it is important that the systemic chemicals should be used when UAV is adopted as the sprayer due to the poor droplet distribution uniformity in the cotton canopy and the application parameters should be optimized at different cotton growth stages before pesticide spraying.

## Supplemental Information

10.7717/peerj.13572/supp-1 Supplemental Information 1 Data of T1-T6-first trial.

10.7717/peerj.13572/supp-2 Supplemental Information 2 Data of T7-T12-first trial.

10.7717/peerj.13572/supp-3 Supplemental Information 3 Data of second trial.

10.7717/peerj.13572/supp-4 Supplemental Information 4 The main physical and flight parameters of the two UAVs used in this work.

10.7717/peerj.13572/supp-5 Supplemental Information 5 Treatments of the first trial on 14/July/2020.

10.7717/peerj.13572/supp-6 Supplemental Information 6 Treatments of the second trial on 22/August/2020.

10.7717/peerj.13572/supp-7 Supplemental Information 7 The environmental data of the field trial experiments.

## Additional Information and Declarations

### Data Availability

The following information was supplied regarding data availability: The raw measurements are available in the Supplemental Files.
