# Supplementary material for: Droplet distribution in cotton canopy using single-rotor and four-rotor unmanned aerial vehicles
Source: PeerJ. 2022 Jun 14;10:e13572. doi: 10.7717/peerj.13572 (PMC9205310; doi:10.7717/peerj.13572)
Supplement: Supplemental Information 4 [file peerj-10-13572-s004.docx]

| Name | Values | |
| --- | --- | --- |
|  | Four-rotor UAV 3WQFTX-10 | Single-rotor UAV 3WQF120-12 |
| Rotor diameter/ mm | 1520 | 3400 |
| Nozzle type | Flat-fan, Lu120-015 | Flat-fan, Lu120-015 |
| No. of Nozzle | 4 | 3 |
| Nozzle flow rate/ Lmin^-1^ | 1.2~3.2 | 1.5~3.0 |
| Nozzle installation | Under the center of rotor | Under spraying boom |
| Tank volume/ L | 10 | `12 |
| Flight velocity/ m s^-1^ | 1~5 | 1~7 |
| Spray swath /m | 3~4 (depends on flight height) | 4~6(depends on flight height) |
| Boom length /m | - | 2 |
| Flight height /m | 1~4 | 1~4 |
