# Supplementary material for: Droplet distribution in cotton canopy using single-rotor and four-rotor unmanned aerial vehicles
Source: PeerJ. 2022 Jun 14;10:e13572. doi: 10.7717/peerj.13572 (PMC9205310; doi:10.7717/peerj.13572)
Supplement: Supplemental Information 5 [file peerj-10-13572-s005.docx]

| **Treatments** | **UAV** | **Flight height (m)** | **Forward mode** |
| --- | --- | --- | --- |
| T1 | Single-rotor | 1 | Head forward |
| T 2 | Single-rotor | 2 | Head forward |
| T 3 | Single-rotor | 3 | Head forward |
| T 4 | Single-rotor | 1 | Tail forward |
| T 5 | Single-rotor | 2 | Tail forward |
| T 6 | Single-rotor | 3 | Tail forward |
| T 7 | Four-rotor | 1 | Head forward |
| T 8 | Four-rotor | 2 | Head forward |
| T 9 | Four-rotor | 3 | Head forward |
| T 10 | Four-rotor | 1 | Tail forward |
| T 11 | Four-rotor | 2 | Tail forward |
| T 12 | Four-rotor | 3 | Tail forward |
