# Supplementary material for: Droplet distribution in cotton canopy using single-rotor and four-rotor unmanned aerial vehicles
Source: PeerJ. 2022 Jun 14;10:e13572. doi: 10.7717/peerj.13572 (PMC9205310; doi:10.7717/peerj.13572)
Supplement: Supplemental Information 6 [file peerj-10-13572-s006.docx]

| Treatments | Flight height (m) | Forward mode | Spraying volume (L ha^-1^) |
| --- | --- | --- | --- |
| T1 | 1 | Head forward | 12.0 |
| T 2 | 1 | Tail forward | 12.0 |
| T 3 | 2 | Head forward | 12.0 |
| T 4 | 2 | Tail forward | 12.0 |
| T 5 | 1 | Head forward | 22.5 |
| T 6 | 1 | Tail forward | 22.5 |
| T 7 | 2 | Head forward | 22.5 |
| T 8 | 2 | Tail forward | 22.5 |
