# Supplementary material for: Droplet distribution in cotton canopy using single-rotor and four-rotor unmanned aerial vehicles
Source: PeerJ. 2022 Jun 14;10:e13572. doi: 10.7717/peerj.13572 (PMC9205310; doi:10.7717/peerj.13572)
Supplement: Supplemental Information 7 [file peerj-10-13572-s007.docx]

| Treatments | Wind directions | Wind speed (m s^-1^) | Air temperature (℃) |
| --- | --- | --- | --- |
| The first trial on 14/07/2020 | | | |
| T1 | South wind | 1.0～2.1 | 30.5 |
| T2 | South wind | 0.8～2.5 | 33.0 |
| T3 | Southwest wind | 1.5～2.1 | 34.0 |
| T4 | South wind | 0.7～1.9 | 34.0 |
| T5 | South wind | 1.2～2.0 | 34.5 |
| T6 | South wind | 1.5～2.2 | 36.5 |
| T7 | Southeast wind | 0.7～1.8 | 34.8 |
| T8 | Southeast wind | 0.3～0.8 | 36.8 |
| T9 | South wind | 0.5～1.4 | 33.4 |
| T10 | Southeast wind | 0.7～1.6 | 33.2 |
| T11 | Southeast wind | 1.2～1.6 | 33.9 |
| T12 | South wind | 0.6～0.8 | 33.3 |
| **The second trial on 22/08/2020** | | | |
| T1 | South wind | 0.2～1.0 | 26.6 |
| T2 | South wind | 0.6～1.8 | 27.7 |
| T3 | South wind | 0.7～2.0 | 27.2 |
| T4 | South wind | 0.6～0.9 | 27.9 |
| T5 | South wind | 0.8～1.8 | 28.0 |
| T6 | South wind | 0.4～1.3 | 28.5 |
| T7 | South wind | 0.4～0.8 | 28.1 |
| T8 | Without wind | 0 | 28.8 |
